# Clinical Significance of Serum Soluble Klotho Levels in Sepsis-Associated Encephalopathy: A Single-Center Prospective, Preliminary Study

**DOI:** 10.3390/jcm15093450

**Published:** 2026-04-30

**Authors:** Ali Cetinkaya, Koca Caliskan, Ahmet Bilal Kandemir, Deniz Avci, Sibel Kuzuguden, Hatice Aslan Sirakaya, Abdullah Ilik, Hilal Sipahioglu

**Affiliations:** 1 Department of Internal Medicine, Kayseri City Education and Research Hospital, Kayseri 38010, Türkiye; denav38@gmail.com (D.A.); hasirakaya@gmail.com (H.A.S.); abdullah_ilik@hotmail.com (A.I.); 2 Department of Internal Medicine, Division of Intensive Care, Kayseri City Hospital, Kayseri 38010, Türkiye; kocaclskn@hotmail.com (K.C.); a.b.kandemir63@gmail.com (A.B.K.); hilalgul1983@gmail.com (H.S.); 3 Department of Biochemistry, Kayseri City Hospital, Kayseri 38010, Türkiye; skuzuguden@hotmail.com

**Keywords:** sepsis-associated encephalopathy, klotho protein, neuroinflammation, intensive care, critical care

## Abstract

**Background/Objectives:** Sepsis-associated encephalopathy (SAE) is an acute brain dysfunction during sepsis with high mortality. Klotho protein is notable for its identified neuroprotective effects in chronic neurodegenerative diseases. This study evaluated temporal changes in serum soluble Klotho levels and their association with clinical recovery in SAE. **Methods:** In this prospective observational study, 750 intensive care unit (ICU) patients were screened and 42 patients with SAE were included. Serum soluble Klotho levels, inflammatory markers, and Glasgow Coma Scale (GCS) scores were recorded on days 1 and 3. Associations between changes in Klotho levels and clinical and inflammatory parameters were analyzed. **Results:** The median GCS score increased from 11 (IQR: 10–13) on day 1 to 12 (IQR: 10–13) on day 3 (*p* < 0.001). Serum soluble Klotho levels decreased significantly from 8114.5 ± 3515.7 pg/mL on day 1 to 6452.9 ± 3390 pg/mL on day 3 (*p* < 0.001). Inflammatory markers, including C-reactive protein and procalcitonin, also showed significant reductions over time (*p* < 0.001). A moderate negative correlation was observed between changes in Klotho levels and GCS scores (r = −0.56, *p* < 0.001). Changes in inflammatory markers were not significantly correlated with Klotho dynamics. **Conclusions:** Serum soluble Klotho levels decrease in parallel with neurological improvements in sepsis-associated encephalopathy and are significantly associated with changes in GCS scores. These findings suggest that Klotho may represent a potential biomarker of disease trajectory and neurological recovery.

## 1. Introduction

Sepsis is a life-threatening clinical syndrome characterized by organ dysfunction resulting from a dysregulated host response to infection [1]. It affects millions of individuals worldwide each year and is associated with a mortality rate approaching 25% [2]. The systemic inflammatory response in sepsis induces profound hemostatic and microcirculatory disturbances, ultimately contributing to multiple organ dysfunction [3]. Among its complications, sepsis-associated encephalopathy (SAE) represents a frequent and serious manifestation, defined as diffuse cerebral dysfunction occurring in the absence of direct central nervous system infection. SAE has been reported to increase mortality rates to 26–49% [4].

SAE encompasses a wide clinical spectrum ranging from mild delirium to deep coma and may occur without detectable structural brain abnormalities [5,6,7]. Altered consciousness and cognitive impairment constitute its hallmark clinical features. The pathogenesis of SAE is multifactorial and incompletely understood. Mechanisms implicated in SAE include systemic inflammation, microcirculatory impairment, blood–brain barrier disruption, cytokine imbalance, mitochondrial dysfunction, oxidative stress, and sepsis-induced immunosuppression [6,7]. Neuroinflammation appears to play a central role in integrating these processes.

Toll-like receptor (TLR)-mediated signaling pathways are critical regulators of innate immune activation and represent key mediators linking peripheral inflammation to central nervous system dysfunction. Dysregulated TLR signaling has been implicated in the development of sepsis and related organ injury [8]. Recent experimental evidence has demonstrated that brain endothelial cells and microglia modulate the release of inflammatory mediators through TLR-dependent pathways, further supporting the contribution of TLR activation to the pathogenesis of SAE [9].

Despite the increasing recognition of SAE as a major contributor to sepsis-related morbidity and mortality, reliable biomarkers reflecting neuroinflammatory dynamics and neurological recovery remain limited in clinical practice. Identifying molecules that may reflect both neuroprotective responses and disease trajectory could improve pathophysiological understanding and prognostic assessment. Klotho protein is a pleiotropic molecule involved in multiple metabolic and cellular regulatory pathways. It is highly expressed in the central nervous system, particularly in the choroid plexus and neurons, and has been associated with remyelination, synaptic function, and cognitive performance [10]. Growing evidence supports the neuroprotective properties of Klotho [11,12]. The secreted isoform of Klotho regulates neuronal activity and has been implicated in aging-related neurodegenerative processes [13]. In addition to the choroid plexus, Klotho expression has been demonstrated in Purkinje cells, hippocampal neurons, and cerebral white matter tissue [14]. Collectively, these findings suggest that Klotho may serve as a critical modulator of neuroinflammatory and neurodegenerative pathways. Despite its recognized neuroprotective properties, the role and temporal dynamics of soluble Klotho in SAE remain largely unexplored.

Therefore, the present prospective study aimed to evaluate temporal changes in serum soluble Klotho levels in patients with SAE and to investigate their association with clinical recovery and neurological outcomes.

## 2. Materials and Methods

This prospective observational study was conducted between 1 April 2025 and 1 September 2025 in the Internal Medicine Intensive Care Unit of Kayseri City Hospital, a regional tertiary referral center. The study was conducted in accordance with the Declaration of Helsinki, and the study protocol was approved by the Non-Interventional Clinical Research Ethics Committee of Kayseri City Hospital (approval date: 11 March 2025; decision number: 360).

Sample size was determined a priori using power analysis to detect temporal changes in serum soluble Klotho levels between day 1 and day 3. Based on prior methodological considerations and pilot observations, a medium effect size (Cohen’s d = 0.50) was assumed [15,16]. For a two-sided paired comparison with α = 0.05 and 80% statistical power, the minimum required sample size was calculated as 34 patients; to compensate for potential data loss, 42 patients were ultimately included.

During the study period, 750 consecutive ICU admissions were screened. Patients without sepsis (*n* = 370) and those meeting predefined exclusion criteria were excluded, including neurodegenerative diseases preventing reliable Glasgow Coma Scale (GCS) assessment (*n* = 48), structural central nervous system pathology (intracranial mass, hemorrhage, or infarction) (*n* = 26), central nervous system or meningeal infections (*n* = 18), acute kidney injury (*n* = 65), electrolyte disturbances (*n* = 63), disorders of glucose metabolism (*n* = 33), hepatic or toxic-metabolic encephalopathy (*n* = 48), pregnancy (*n* = 1), refusal to participate (*n* = 28), and incomplete follow-up (*n* = 8). Consequently, 42 patients with sepsis who developed sepsis-associated encephalopathy during ICU stay were included in the final analysis.

**Figure 1:**
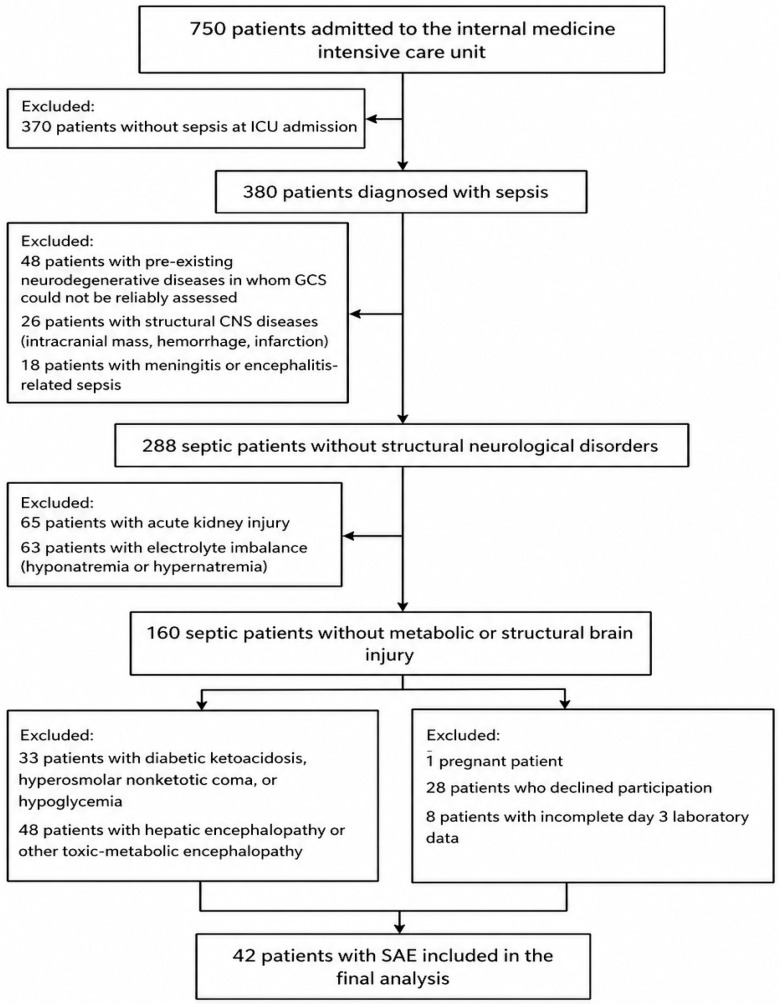
Flowchart of patient selection and exclusion criteria.

Sepsis-associated encephalopathy was defined as an acute alteration in mental status ranging from delirium to coma in the presence of sepsis, in the absence of structural brain lesions and after exclusion of other metabolic, toxic, or primary neurological causes [17]. The exclusion process is illustrated in Figure 1.

Delirium screening was routinely performed twice daily in our intensive care unit using the Confusion Assessment Method for the ICU (CAM-ICU). In accordance with standard delirium assessment protocols, patients were first evaluated for the level of consciousness using the Richmond Agitation Sedation Scale (RASS), and delirium assessment was performed only in patients with a RASS score ≥ −3. For the purpose of the present study, patients included in the SAE group demonstrated comparable clinical features consistent with acute brain dysfunction, including acute changes in mental status, fluctuating course, and inattention as assessed during routine CAM-ICU evaluations. Because only six patients required mechanical ventilation during the first three days of follow-up, sedation levels were minimal in the majority of the cohort, and additional stratification according to RASS scores was not considered clinically informative for the primary biomarker analysis. Consistent with previous literature, SAE was considered in septic patients presenting with acute alterations in mental status (including delirium or decreased level of consciousness) without an alternative neurological cause. This approach aligns with current descriptions of the spectrum of sepsis-associated encephalopathy ranging from delirium to coma [17].

Serum soluble Klotho levels were measured using a commercially available human Klotho ELISA kit (Reed Biotech, Shanghai, China; catalog no. RE2580H) according to the manufacturer’s instructions. Blood samples were centrifuged at 3000 rpm for 4 min, aliquoted, and immediately stored at −80 °C until analysis. Two frozen serum aliquots of 0.35 mL each (total 0.7 mL per patient) were stored in 1.5 mL Eppendorf tubes (Eppendorf SE, Hamburg, Germany).

The assay detection range was 320–20,000 pg/mL with an analytical sensitivity of 190 pg/mL. Measurements were performed in triplicate using the sandwich ELISA method on microplates pre-coated with antibodies specific to human Klotho protein. Sample concentrations were calculated from calibration curves generated using standards with known concentrations, demonstrating excellent linearity (regression coefficient r = 0.998). The intra-assay and inter-assay coefficients of variation were both <5%, indicating high analytical precision and reproducibility. Final concentrations were expressed in pg/mL in accordance with the assay reporting system.

Demographic characteristics (age and sex), ICU and hospital length of stay, need for mechanical ventilation and vasopressor support, and mortality were recorded. Disease severity was assessed using the Acute Physiology and Chronic Health Evaluation II (APACHE II) score, the Sequential Organ Failure Assessment (SOFA) score, and the modified Nutrition Risk in the Critically Ill (mNUTRIC) score (without IL-6). Neurological status was evaluated using the Glasgow Coma Scale (GCS) on day 1 (admission) and day 3.

Blood samples were collected on days 1 and 3 of sepsis to evaluate early temporal dynamics during the acute phase of sepsis-associated encephalopathy, as SAE is characterized by an early onset of brain dysfunction during sepsis, ranging from delirium to coma [17]. Serum soluble Klotho levels were measured together with inflammatory markers (C-reactive protein and procalcitonin), renal function tests (blood urea nitrogen and estimated glomerular filtration rate), hepatic enzymes (aspartate aminotransferase, alanine aminotransferase, and total bilirubin), hematological parameters (white blood cell count, lymphocyte count, platelet count, erythrocyte count, and mean platelet volume), coagulation parameters (international normalized ratio, fibrinogen, and D-dimer), metabolic markers (lactate and uric acid), lactate dehydrogenase as a tissue injury marker, and relevant clinical scores.

Changes in Klotho levels were calculated as the difference between day 3 and day 1 values (ΔKlotho). Similarly, delta changes were calculated for GCS, C-reactive protein, procalcitonin, and white blood cell counts.

Statistical analyses were performed using SPSS Statistics software version 22 (IBM Corp., New York, NY, USA). The distribution of continuous variables was assessed using the Shapiro–Wilk test. Normally distributed data are presented as mean ± standard deviation, whereas non-normally distributed data are presented as median (IQR). Categorical variables are expressed as number and percentage.

Comparisons between day 1 and day 3 values were performed using the paired t-test for normally distributed variables and the Wilcoxon signed-rank test for non-normally distributed variables. Associations between serum Klotho levels and clinical and inflammatory parameters were evaluated using Spearman’s correlation analysis.

A two-sided *p*-value < 0.05 was considered statistically significant. The strength of correlations was interpreted as follows: absolute correlation coefficients of 0.00–0.29 were considered weak, 0.30–0.59 moderate, and ≥0.60 strong.

## 3. Results

The mean age of patients with SAE was 73.14 ± 13.57 years, and 45% were male. The median ICU length of stay was 8 days (3–76), and the median hospital length of stay was 17.5 days (2–120). The median baseline GCS score was 11 (IQR: 10–13), the APACHE II score was 22.98 ± 7.88, and the NUTRIC score was 5.38 ± 1.37. During ICU stay, 14% of patients required mechanical ventilation, 17% required vasopressor support, and ICU mortality was 26%. The median SOFA score increased from 3 (IQR: 0–10) at hospital admission to 5 (IQR: 4–7) at the time of SAE diagnosis, indicating progression of organ dysfunction prior to the onset of neurological involvement (Table 1).

Serum soluble Klotho levels significantly decreased from day 1 to day 3 (8114.5 ± 3515.7 pg/mL vs. 6452.9 ± 3390 pg/mL, *p* < 0.001). In parallel, a significant increase in GCS scores was observed (11 (10–13) vs. 12 (10–13), *p* < 0.001).

A marked reduction in inflammatory markers was detected over time. CRP levels significantly decreased from day 1 to day 3 (177.90 ± 98.68 mg/L vs. 108.89 ± 73.72 mg/L, *p* < 0.001). Procalcitonin levels also showed a significant decrease in median values (7.04 [0.16–42.80] vs. 0.70 [0.05–29.10], *p* < 0.001).

Renal function parameters, including glomerular filtration rate (GFR) (day 1: 70.92 ± 7.28; day 3: 72.47 ± 19.40; *p* = 0.125) and blood urea nitrogen (BUN) (day 1: 40.59 ± 17.42; day 3: 40.19 ± 14.57; *p* = 0.901), did not show significant differences between the two time points. Similarly, no significant changes were observed in aspartate aminotransferase (AST) (*p* = 0.903), alanine aminotransferase (ALT) (*p* = 0.272), total bilirubin (*p* = 0.142), or uric acid levels (*p* = 0.854).

Among hematological parameters, only red blood cell counts significantly decreased on day 3 (*p* = 0.047), whereas white blood cell count, lymphocyte count, platelet count, and mean platelet volume (MPV) did not change significantly. No significant differences were observed in coagulation parameters, including international normalized ratio (INR), fibrinogen, and D-dimer levels between day 1 and day 3 (*p* = 0.258, *p* = 0.663, and *p* = 0.662, respectively).

LDH levels significantly increased on day 3 (329.93 ± 124.79 U/L vs. 416.00 ± 305.46 U/L, *p* = 0.027). In contrast, lactate levels significantly decreased (1.95 ± 1.29 vs. 1.48 ± 1.32 mmol/L, *p* = 0.048). Day 1 and day 3 clinical and laboratory parameters are summarized in Table 2.

**Figure 2:**
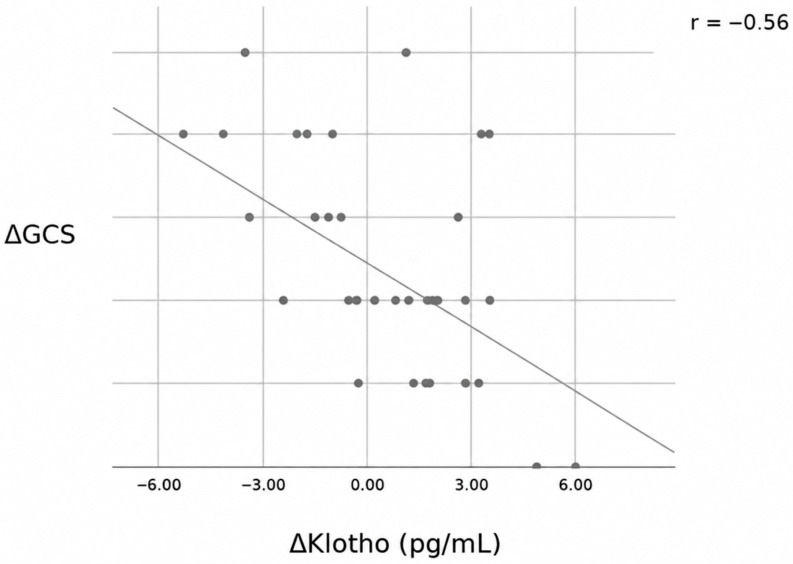
Correlation between changes in Glasgow Coma Scale (GCS) score and serum soluble Klotho levels. The solid line represents the linear regression line (r = −0.56, *p* < 0.001).

**Figure 3:**
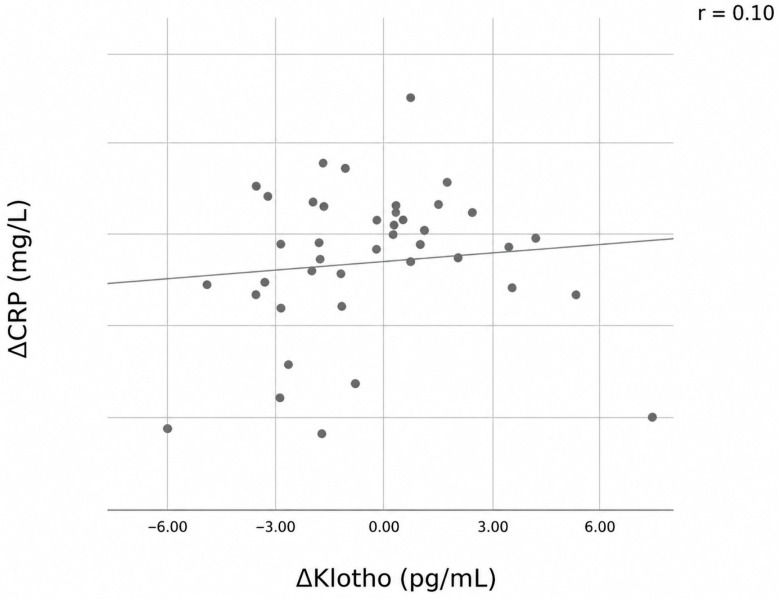
Correlation between changes in C-reactive protein (CRP) levels and serum soluble Klotho levels. The solid line represents the linear regression line (r = 0.10, *p* = 0.524).

A moderate and significant negative correlation was observed between changes in serum soluble Klotho levels (ΔKlotho) and changes in GCS scores (ΔGCS) (r = −0.56, *p* < 0.001) (Figure 2). In contrast, correlation analysis revealed a weak and non-significant association between changes in CRP levels and changes in Klotho levels (ΔCRP vs. ΔKlotho; r = 0.10, *p* = 0.524) (Figure 3). Similarly, changes in procalcitonin levels were not significantly correlated with changes in Klotho levels (*p* = 0.548).

In univariable analyses, only ΔKlotho was significantly associated with ΔGCS, whereas inflammatory markers, severity scores, and age were not. In multivariable linear regression analysis adjusting for SOFA score and CRP, ΔKlotho remained independently associated with ΔGCS (β = 0.543, *p* < 0.001). Neither SOFA score nor CRP showed a significant association with ΔGCS in the adjusted model (Table 3). The model explained approximately 34% of the variance in ΔGCS (R^2^ = 0.340).

Univariable logistic regression analysis for mortality is provided in Supplementary Table S1. Additional correlation analyses between ΔKlotho, ΔGCS, ΔCRP, and changes in laboratory and clinical parameters are presented in Supplementary Tables S2–S4 and S6. The components of the clinical severity scores used in the study are provided in Supplementary Table S5. The SOFA score calculated at the time of SAE diagnosis is presented in Supplementary Table S7, while Table 3 reflects SOFA values at hospital admission; all other variables and model parameters remain identical.

## 4. Discussion

This prospective study is, to our knowledge, among the first to evaluate serum Klotho levels together with neurological status in sepsis-associated encephalopathy and to provide clinical evidence in this context. The negative correlation between Klotho levels and GCS scores and the independent course of Klotho from CRP support the hypothesis that Klotho may function as a neuroprotective factor that is dynamically regulated during acute neuroinflammatory processes and may constitute a biologically relevant candidate molecule.

Our findings indicate that the Klotho protein does not appear to behave like a classical acute-phase reactant and follows a course that seems to be largely independent of hepatic and renal function.

When comparing day 1 and day 3 in our study, GCS scores were lower on day 1 and increased on day 3, whereas Klotho levels were higher on day 1 and decreased on day 3 but remained elevated compared with the normal population. The negative correlation between ΔKlotho and ΔGCS suggests that Klotho is dynamically regulated during the course of neurological recovery. However, this observation should be interpreted with caution and considered hypothesis-generating, as causal relationships cannot be inferred from this observational design. These findings suggest that Klotho may participate in biological processes associated with neurological recovery in SAE. Importantly, the association between changes in Klotho levels and neurological recovery persisted after adjustment for disease severity and inflammatory markers, suggesting that this relationship is not solely explained by general illness severity or systemic inflammation.

In the general population, serum soluble Klotho levels demonstrate a wide distribution, ranging from approximately 60 pg/mL to over 3300 pg/mL, although most reports describe concentrations between 200 and 1600 pg/mL with mean values around 500–800 pg/mL [15,18,19,20,21,22]. This variability has been attributed to individual biological factors, assay-related variability, and methodological differences in measurement techniques, and circulating Klotho levels are also known to decline with age. In these studies, measurements were commonly obtained using sandwich ELISA-based assays comparable to the method applied in our investigation.

In the present cohort of patients with sepsis-associated encephalopathy, serum Klotho concentrations were substantially higher than these reference values, reaching 8114.5 ± 3515.7 pg/mL on day 1 and 6452.9 ± 3390.0 pg/mL on day 3. Markedly elevated circulating Klotho concentrations have also been described in specific disease states. For example, sarcoidosis cohorts have shown mean serum Klotho levels of approximately 5300 pg/mL [23], and patients with acromegaly have demonstrated pre-treatment concentrations near 4200 pg/mL [24], values that clearly exceed typical reference intervals derived from healthy populations. These observations suggest that Klotho may undergo disease-specific upregulation in response to inflammatory, endocrine, or systemic stress conditions.

Within this context, the elevated serum Klotho concentrations observed in SAE may reflect an acute neuroprotective or stress-responsive biological activation rather than solely analytical variability. Moreover, the temporal decline in Klotho levels occurring in parallel with improvement in Glasgow Coma Scale scores supports the interpretation that Klotho dynamics may be linked to neurological recovery processes, potentially through mechanisms involving consumption, redistribution, or clearance during the resolution of acute neuroinflammation. Given known inter-assay differences and population heterogeneity across studies, absolute concentration comparisons should be interpreted cautiously, and the principal significance of our findings lies in the consistent within-patient temporal change in Klotho levels accompanying clinical improvement.

In recent clinical cohorts of SAE, the demographic profile generally consists of elderly populations, with studies reporting either male or female predominance. Yupeng Han et al. reported a median age of 76 years with a male predominance of 56% [25]. Zhang et al. reported a female predominance of 57.6% [26], whereas a meta-analysis of 13 cohort studies demonstrated a slight male predominance [27]. In our study, the mean age (73.14 years) was consistent with the literature, and a slight female predominance was observed (55%).

In our cohort, the median hospital length of stay was 17 days and the median ICU length of stay was 8 days. These durations are consistent with the upper range reported in large cohort studies and are shorter than the prolonged lengths of stay reported in neurocritical SAE populations, reflecting the clinical heterogeneity of SAE and variability in disease severity [5,27,28,29]. Jiayu et al. reported a mean ICU stay of 3.3–3.9 days [30], whereas Shen et al. reported mean ICU lengths of stay of 9.5–11.2 days [28]. A previous regional study reported a mean ICU stay of 9 days and a mean hospital stay of 15 days [31]. Disease severity, clinical course, and inclusion criteria may influence hospitalization duration, and our cohort appears comparable to studies reporting longer lengths of stay.

In a regional cohort of 254 geriatric patients with sepsis or septic shock, a mortality of 48% was reported [31]. Another regional study reported mortality up to 51% in sepsis patients with a mean GCS of 13 [32]. Zhao et al. reported in-hospital mortality of 12.7% in a large cohort of 22,361 patients [29]. Han et al. further demonstrated significantly higher mortality in sepsis patients who developed SAE compared with those without SAE (28.78% vs. 12.59%, *p* < 0.001) [25]. Consistently, previous SAE cohorts have shown APACHE II values around 22 in non-survivors with overall 28-day mortality near 33% [33], whereas septic ICU populations with median APACHE II scores near 27 have demonstrated mortality approaching 49% [34]. The ICU mortality observed in our cohort (26%) is therefore comparable with previously reported SAE populations and suggests a similar clinical severity profile.

Klotho is a recently identified protein involved in multiple metabolic pathways and is highly expressed in the choroid plexus and neurons, playing an important role in remyelination and cognitive processes [10]. The high Klotho levels observed on day 1 and their decline on day 3 are consistent with the hypothesis that Klotho may be upregulated during the early phase of SAE and subsequently consumed during clinical recovery.

Wu et al. investigated the relationship between Klotho levels and cognitive function using Mendelian randomization and cross-sectional analyses. Although Mendelian randomization did not demonstrate a causal association with dementia, observational analyses showed a significant correlation with cognitive function [35]. Torbus et al. suggested neuroprotective effects of Klotho in neurodegenerative diseases [13], and Jiang et al. reported that low Klotho levels were associated with increased all-cause mortality in depression [22].

Participants with higher Klotho levels have been reported to have a lower risk of all-cause mortality. Additionally, in vitro models mimicking chronic kidney disease conditions have shown that Klotho may reduce cellular inflammatory responses and improve cellular lipid metabolism [36]. Bugoslowa et al. reported that Klotho regulates metabolic homeostasis through fibroblast growth factors (FGF19, FGF21, and FGF23) and exerts neuroprotective, cardioprotective, and antitumor effects [37]. Considering the pathophysiology of septic encephalopathy, these metabolic and cellular protective effects may contribute to limiting central nervous system injury.

Cararo-Lopes et al. suggested that the brain Klotho pool plays a critical role in supporting myelination and maintaining healthy brain aging [12]. Abraham et al. demonstrated that Klotho is a pleiotropic protein released into the cerebrospinal fluid via A Disintegrin and Metalloproteinases (ADAM10 and ADAM17), supporting neuroprotection and cognitive function [38,39]. Chen et al. proposed that transcriptional approaches to enhance Klotho activity may represent novel therapeutic modalities in the future [40]. Our findings are consistent with these studies and suggest that Klotho may exert protective effects not only in chronic diseases but also in acute cognitive dysfunction such as SAE.

Previous studies have reported an inverse correlation between serum Klotho levels and inflammatory markers, including CRP, suggesting an anti-inflammatory role for Klotho [41,42,43]. However, in our study, no association was observed between Klotho levels and CRP. Although our study evaluated short-term changes in Klotho levels over a three-day period, which may limit the ability to fully capture its relationship with inflammatory parameters, these findings suggest that Klotho may reflect specific neuroinflammatory mechanisms rather than systemic inflammatory burden in SAE and may increase rapidly during acute neurological alterations. Notably, traditional markers of disease severity, including SOFA score and inflammatory markers such as CRP, were not associated with ΔGCS in our cohort, whereas ΔKlotho showed a consistent and independent association with neurological recovery. In supplementary analyses, ΔKlotho showed significant negative correlations with changes in liver enzymes (AST and ALT). However, AST and ALT levels remained within or close to normal reference ranges in our cohort, and the clinical significance of these associations remains uncertain. These findings may reflect broader systemic physiological responses rather than direct hepatic involvement.

In the study by Lanzani et al., Klotho was reported to exert anti-inflammatory and antioxidant effects, regulate mineral metabolism via FGF-23, and higher serum Klotho levels were associated with reduced cardiovascular and non-cardiovascular mortality [44]. These findings are consistent with our results regarding the protective role of Klotho; however, unlike that study, our findings suggest that Klotho levels may follow a course independent of systemic inflammatory processes.

Experimental animal and primate studies strongly support the neuroprotective role of Klotho in the central nervous system. In murine models, impaired Klotho gene expression has been associated with shortened lifespan, premature aging phenotypes, and cognitive dysfunction, whereas Klotho overexpression has been reported to extend lifespan [45,46]. In another animal study, peripheral administration of Klotho protein increased M2 microglial polarization, improved cognitive function, and reduced neuroinflammation [47]. Cellular studies suggest that Klotho may regulate macrophage polarization and attenuate age-related inflammation and oxidative stress via the TLR4/MyD88/NF-κB signaling pathway. Studies demonstrating co-localization of Klotho with neuronal marker NeuN and oligodendrocyte marker Olig2, as well as defining its anatomical distribution in brain parenchyma, further support its role in myelin integrity and neuronal structure [11]. In aging studies in rhesus monkeys, white-matter alterations were identified as critical determinants of cognitive decline, and microarray analyses showed that Klotho is among the genes associated with lifespan [14].

Renal clearance has been proposed as one of the potential mechanisms contributing to changes in circulating soluble Klotho levels, particularly in studies conducted in patients with chronic kidney disease where altered urinary Klotho excretion accompanies impaired renal function. Prior evidence demonstrating significant urinary Klotho excretion primarily in patients with stage 2–4 chronic kidney disease supports the notion that clinically meaningful renal elimination is largely confined to populations with reduced renal function [48]. However, this mechanism is less likely to explain the temporal decline observed in the present cohort. Patients with overt renal failure were excluded, and mean glomerular filtration rate values remained within a relatively preserved range, measuring approximately 70 mL/min/1.73 m^2^ on day 1 and 72 mL/min/1.73 m^2^ on day 3. The stability of renal function across the observation period argues against a major contribution of renal clearance to the observed reduction in serum Klotho.

Similarly, hepatic injury-related mechanisms also appear unlikely, as aspartate aminotransferase (AST) and alanine aminotransferase (ALT) levels remained within the normal reference range on both day 1 and day 3, showed no significant temporal change, and demonstrated no association with ΔKlotho levels. Taken together, these findings suggest that the temporal decline in circulating Klotho in sepsis-associated encephalopathy is more plausibly related to disease-specific biological regulation, redistribution, or utilization during neurological recovery rather than altered renal or hepatic handling.

For these reasons, we hypothesize that Klotho upregulation may represent an acute endogenous neuroprotective response to septic brain injury, followed by gradual consumption during neurorecovery. This dynamic pattern suggests that Klotho may participate in neuronal repair mechanisms rather than simply reflecting systemic inflammatory burden.

This study has some limitations. The single-center observational design limits generalizability, and the relatively small sample size (*n* = 42) may have reduced statistical power, particularly for subgroup analyses and multivariable models. The absence of a healthy regional control group prevented determination of local reference ranges for serum Klotho levels and required comparison with previously reported normative data. Although comparisons were made with studies using similar sandwich ELISA-based methodologies, the unexpectedly high serum Klotho concentrations observed in our cohort may partly reflect assay-related variability, inter-individual biological heterogeneity, and the lack of region-specific reference ranges. Therefore, direct comparison with published normative values should be interpreted with caution. The absence of specific neuronal injury biomarkers (e.g., NSE or S100B) limits mechanistic interpretation of Klotho dynamics. Future studies incorporating dedicated neuroinjury markers are warranted. The lack of systematic electroencephalographic (EEG) monitoring is another limitation of this study. EEG was not routinely performed in all patients due to clinical and logistical constraints in the intensive care setting, which may have limited the ability to objectively characterize the neurological features of sepsis-associated encephalopathy.

Although Klotho levels decreased within the first three days and showed a negative correlation with GCS, the lack of association with inflammatory markers may have been influenced by measurement timing, biological variability, and sample size. Study funding was provided by the investigators, reflecting financial and technical infrastructure limitations commonly encountered in biomarker research in developing countries, which may have restricted sample size, repeated measurements, and comprehensive biochemical analyses.

Finally, the observational design precludes causal inference, and larger multicenter prospective controlled studies are needed to clarify the role of Klotho in the pathophysiology of sepsis-associated encephalopathy.

## 5. Conclusions

Sepsis-associated encephalopathy is a complex clinical condition associated with high mortality and morbidity that requires a multidisciplinary diagnostic and therapeutic approach. In this study, serum Klotho levels in patients with SAE were higher than reference values reported in previous studies. The significant decline in Klotho levels from day 1 to day 3 and the negative correlation with GCS suggest that Klotho may be dynamically regulated during acute neurological dysfunction and subsequently decrease in parallel with clinical improvement, potentially reflecting neuroprotective processes.

Overall, these findings indicate that Klotho may represent a dynamically regulated molecule in the pathophysiology of SAE and a potential biomarker of neurological recovery, warranting confirmation in larger prospective, controlled studies.

## Figures and Tables

**Table 1:** Baseline characteristics of patients with sepsis-associated encephalopathy (*n* = 42).

Variable	Total (*n* = 42)
Age (years), mean ± SD	73.14 ± 13.57
Male sex, n (%)	19 (45)
ICU length of stay (days), median (min–max)	8 (3–76)
Hospital length of stay (days), median (min–max)	17.5 (2–120)
PaO_2_ (mmHg), mean ± SD	93.76 ± 1.32
FiO_2_ (%), mean ± SD	37.78 ± 4.66
GCS, median (IQR)	11(10–13)
APACHE II score, mean ± SD	22.98 ± 7.88
NUTRIC score, mean ± SD	5.38 ± 1.37
SOFA score (baseline), median (IQR)	3.0 (0–10)
SOFA score at SAE diagnosis, median (IQR)	5 (4–7)
Mechanical ventilation, n (%)	6 (14)
Vasopressor requirement during ICU stays, n (%)	7 (17)
ICU mortality, n (%)	11 (26)

Data are presented as mean ± standard deviation (SD) or median (min–max); the Glasgow Coma Scale (GCS) score is presented as median (interquartile range). **Abbreviations**: ICU, intensive care unit; PaO_2_, partial pressure of arterial oxygen; FiO_2_, fraction of inspired oxygen; GCS, Glasgow Coma Scale; APACHE II, Acute Physiology and Chronic Health Evaluation II; NUTRIC, Nutrition Risk in the Critically Ill; SOFA, Sequential Organ Failure Assessment.

**Table 2:** Comparison of laboratory and clinical parameters on day 1 and day 3.

Variable	Day 1 (*n* = 42)	Day 3 (*n* = 42)	*p*-Value
Klotho (pg/mL)	8114.5 ± 3515.7	6452.9± 3390	<0.001
GCS median (IQR)	11 (10–13)	12 (10–13)	<0.001
CRP (mg/L)	177.9 ± 98.68	108.89 ± 73.72	<0.001
Procalcitonin (µg/L)	7.04 (0.16–42.8)	0.7 (0.05–29.1)	<0.001
GFR (mL/min/1.73 m^2^)	70.92 ± 7.28	72.47 ± 19.4	0.125
BUN (mg/dL)	40.59 ± 17.42	40.19 ± 14.57	0.901
AST (U/L)	27 (5–42)	29 (5–51)	0.903
ALT (U/L)	21.5 (5–39)	19 (5–47)	0.272
Albumin (g/dL)	3.05 ± 0.49	3.54 ± 0.68	0.496
Uric acid (mg/dL)	6.30 ± 3.12	6.37 ± 3.17	0.854
Total bilirubin (mg/dL)	0.7 (0.2–6.7)	0.7 (0.3–6.4)	0.142
LDH (U/L)	329.93 ± 124.79	416 ± 305.46	0.027
neutrophil (10^3^/µL)	10.48 ± 6.43	10.05 ± 7.52	0.609
MPV (fL)	10.63 ± 1.21	10.77 ± 1.91	0.253
RBC (10^6^/µL)	3.87 ± 0.82	3.71 ± 0.76	0.047
Lymphocytes (10^3^/µL)	1.23 ± 1.23	1.04 ± 0.76	0.302
Platelets (10^3^/µL)	188.16 ± 114.7	175.07 ± 97.27	0.193
INR	1.44 ± 0.76	1.32 ± 0.37	0.258
Fibrinogen (mg/L)	4045.10 ± 1538.37	4127.27 ± 1371.02	0.663
D-dimer (ng/mL)	10,317.5 ± 7255.74	10,357.50 ± 5415.42	0.662
Lactate (mmol/L)	1.95 ± 1.29	1.48 ± 1.32	0.048

Data are presented as mean ± standard deviation (SD) for normally distributed variables and median (min–max) or median (interquartile range, IQR) for non-normally distributed variables. *p*-values were calculated using paired t-test or Wilcoxon signed-rank test, as appropriate. **Abbreviations**: MPV, mean platelet volume; RBC, red blood cell count; CRP, C-reactive protein; LDH, lactate dehydrogenase; INR, international normalized ratio.

**Table 3:** Univariable and Multivariable Linear Regression Analysis for Factors Associated with ΔGCS.

Variables	Univariable β (95% CI)	*p*	Multivariable β (95% CI)	*p*
ΔKlotho	0.255 (0.134–0.376)	<0.001	0.247 (0.119–0.375)	<0.001
SOFA	0.084 (−0.096–0.264)	0.352	0.104 (−0.049–0.257)	0.177
CRP	−0.002 (−0.006–0.002)	0.226	−0.001 (−0.005–0.003)	0.600
Age	−0.001 (−0.030–0.028)	0.948		
APACHE II	0.005 (−0.047–0.057)	0.847		
Procalcitonin	0.002 (−0.037–0.041)	0.924		

β: unstandardized regression coefficient; CI: confidence interval; Δ: change between measurements; GCS: Glasgow Coma Scale; SOFA: Sequential Organ Failure Assessment; CRP: C-reactive protein; APACHE II: Acute Physiology and Chronic Health Evaluation II. Linear regression analyses were used for both univariable and multivariable models. Clinically relevant variables were included in the multivariable analysis regardless of univariable significance. A two-sided *p* value < 0.05 was considered statistically significant. Note: In this table, the SOFA score reflects values at hospital admission. The SOFA score calculated at the time of SAE diagnosis is provided in Supplementary Table S7. All other variables and model parameters remain unchanged.

## Data Availability

The data presented in this study are available on reasonable request from the corresponding author. The data are not publicly available due to privacy and ethical restrictions.

## Supplementary Materials

The following supporting information can be downloaded at https://www.mdpi.com/article/10.3390/jcm15093450/s1. Supplementary Table S1: Univariable mortality analysis; Supplementary Table S2: Correlation between ΔKlotho and changes in laboratory and clinical parameters; Supplementary Table S3: Correlation between ΔGCS and changes in laboratory and clinical parameters; Supplementary Table S4: Correlation between ΔCRP and changes in laboratory and clinical parameters; Supplementary Table S5: Components of Clinical Severity Scores Used in the Study; Supplementary Table S6. Correlation Analysis Between Changes in Klotho Levels and Biochemical Parameters; Supplementary Table S7. Univariable and Multivariable Linear Regression Analysis for Factors Associated with ΔGCS, Including SOFA Score at the Time of SAE Diagnosis.

## Abbreviations

The following abbreviations are used in this manuscript:

ADAM	A Disintegrin and Metalloproteinase
ALT	Alanine Aminotransferase
APACHE II	Acute Physiology and Chronic Health Evaluation II
AST	Aspartate Aminotransferase
AUC	Area Under the Curve
BUN	Blood Urea Nitrogen
CRP	C-Reactive Protein
ELISA	Enzyme-Linked Immunosorbent Assay
FGF	Fibroblast Growth Factor
FiO_2_	Fraction of Inspired Oxygen
GCS	Glasgow Coma Scale;
GFR	Glomerular Filtration Rate
ICU	Intensive Care Unit
INR	International Normalized Ratio
IQR	Interquartile Range
LDH	Lactate Dehydrogenase
MPV	Mean Platelet Volume
NF-κB	Nuclear Factor Kappa B
NUTRIC	Nutrition Risk in the Critically Ill
PaO_2_	Partial Pressure of Arterial Oxygen
RBC	Red Blood Cell
ROC	Receiver Operating Characteristic
SAE	Sepsis-Associated Encephalopathy
SD	Standard Deviation
SOFA	Sequential Organ Failure Assessment
TLR	Toll-Like Receptor
