# Supplementary material for: Clinical Significance of Serum Soluble Klotho Levels in Sepsis-Associated Encephalopathy: A Single-Center Prospective, Preliminary Study
Source: J Clin Med. 2026 Apr 30;15(9):3450. doi: 10.3390/jcm15093450 (PMC13164086; doi:10.3390/jcm15093450)
Supplement: Supplementary file 1 [file jcm-15-03450-s001.zip › jcm-4228663-supplementary.pdf]

Supplementary Table S1. Univariate Logistic Regression Analysis for Mortality in the Serum Soluble Klotho Study

Variable	P	OR	95% CI Lower	95% CI Upper
SOFA	0.421	1.131	0.838	1.527
GCS Day-1	0.237	0.891	0.737	1.079
GCS Day-3	0.080	0.831	0.675	1.023
APACHE II	0.147	1.066	0.978	1.163
GCS Δ	0.136	0.813	0.620	1.067
Klotho Day-1	0.139	1.139	0.972	1.323
Klotho Day-3	0.106	1.024	0.789	1.254
Klotho Δ	0.624	1.016	0.950	1.083
CRP Δ	0.266	0.997	0.997	1.004

Notes: Univariable logistic regression analysis was performed to evaluate the association between clinical variables and mortality. Odds ratios (ORs) with 95% confidence intervals (CIs) are presented. A two-sided p-value < 0.05 was considered statistically significant.

Supplementary Table S2. Correlations between ΔKlotho and laboratory/clinical parameter changes

Variable (Δ)	r	p-value
GCS	**−0.564**	**<0.001**
CRP	0.102	0.524
AST	0.511	0.001
ALT	0.341	0.027
Total bilirubin	0.280	0.088
Neutrophil count	−0.012	0.938
Lymphocyte count	0.030	0.853
Platelet count	−0.028	0.863
INR	−0.117	0.461

Notes: Correlations were assessed using Pearson correlation analysis. Correlation coefficients (r) and corresponding two-sided p-values are presented. A p-value < 0.05 was considered statistically significant. Δ indicates change between day 1 and day 3.

Supplementary Table S3. Correlations between ΔGCS and laboratory parameter changes

Variable (Δ)	r	p-value
Klotho	**−0.564**	**<0.001**
CRP	−0.157	0.327
AST	−0.012	0.941
ALT	0.081	0.629
Total bilirubin	−0.302	0.066
Neutrophil count	0.044	0.787
Lymphocyte count	0.099	0.537
Platelet count	−0.267	0.091
INR	−0.060	0.709

Notes: Correlations were assessed using Pearson correlation analysis. Correlation coefficients (r) and corresponding two-sided p-values are presented. A p-value < 0.05 was considered statistically significant. Δ indicates change between day 1 and day 3.

Supplementary Table S4. Correlations between ΔCRP and laboratory/clinical parameter changes

Variable (Δ)	r	p-value
Klotho	0.102	0.524
GCS	−0.157	0.327
AST	0.549	<0.001
ALT	0.081	0.629
Total bilirubin	0.375	0.020
Neutrophil count	0.236	0.133
Lymphocyte count	0.256	0.102
Platelet count	0.127	0.422
INR	0.122	0.443

Notes: Correlations were assessed using Pearson correlation analysis. Correlation coefficients (r) and corresponding two-sided p-values are presented. A p-value < 0.05 was considered statistically significant. Δ indicates change between day 1 and day 3.

Supplementary Table S5. Components of Clinical Severity Scores Used in the Study

Score	Variables Included
APACHE II	Age; temperature; mean arterial pressure; heart rate, respiratory rate; oxygenation (PaO_2 or A–a gradient); arterial pH, serum sodium; serum potassium; serum creatinine; hematocrit; white blood cell count; Glasgow Coma Scale
SOFA	PaO_2/FiO_2 ratio; platelet count; serum bilirubin; mean arterial pressure or vasopressor requirement; Glasgow Coma Scale; serum creatinine or urine output
Modified NUTRIC (mNUTRIC)	Age; APACHE II score; SOFA score; number of comorbidities; days from hospital admission to ICU admission

Supplementary Table S6. Correlation Analysis Between Changes in Klotho Levels and Biochemical Parameters

**Variable**	**ΔKlotho correlation (ϱ)**	**p**
ASTdelta	-0.438	0.004
ALTdelta	-0.350	0.023

Supplementary Table S7. Univariable and Multivariable Linear Regression Analysis for Factors Associated with ΔGCS, Including SOFA Score at the Time of SAE Diagnosis

**Variables**	Univariable β (95% CI)	**p**	Multivariable β (95% CI)	**p**
ΔKlotho	0.255 (0.134–0.376)	**<0.001**	0.247 (0.118–0.376)	**<0.001**
SOFA	-0.039 (-0.229–0.150)	0.678	-0.015 (-0.180–0.149)	0.853
CRP	−0.002 (−0.006–0.002)	0.226	-0.001 (-0.004–0.003)	0.676
Age	−0.001 (−0.030–0.028)	0.948		
APACHE II	0.005 (−0.047–0.057)	0.847		
Procalcitonin	0.002 (−0.037–0.041)	0.924		

Data are presented as unstandardized regression coefficients (β) with 95% confidence intervals (CI). Δ indicates change between measurements. GCS: Glasgow Coma Scale; SOFA: Sequential Organ Failure Assessment; CRP: C-reactive protein; APACHE II: Acute Physiology and Chronic Health Evaluation II. SOFA score was calculated at the time of SAE diagnosis using contemporaneous clinical and laboratory parameters. Variables included in the multivariable model were selected based on clinical relevance. A two-tailed p value < 0.05 was considered statistically significant.
